# Three-dimensional stability analysis of tunnel face based on unified strength theory

**DOI:** 10.1038/s41598-023-39554-z

**Published:** 2023-07-29

**Authors:** Qiao Liang, Junjie Xu, Yuanguo Wei

**Affiliations:** 1 Department of Construction Engineering, Hunan Institute of Engineering, Xiangtan, 411104 Hunan China; 2 Hunan Provincial Key Laboratory of Intelligent Disaster Prevention-Mitigation and the Ecological Restoration in Civil Engineering, Hunan Institute of Engineering, Xiangtan, 411104 Hunan China

**Keywords:** Civil engineering, Mechanical engineering

## Abstract

The impact of cyclic footage and intermediate principal stress on the stability of the tunnel-face area are analyzed in this study using the theory of limit analysis. The study introduces the unified strength theory and proposes three-dimensional logarithmic spiral failure modes with corresponding velocity fields. The influence of various parameters on the tunnel-face area stability is analyzed, and it is found that when the internal friction angle is less than 30°, the internal friction angle parameters should be improved first to enhance stability, while when the internal friction angle is greater than 30°, cohesion should be prioritized. When using the double shear uniform strength theory in the tunnel-face area, the intermediate principal stress can improve the stability of the tunnel face. Results show that Mohr–Coulomb criterion calculations are conservative in the good surrounding ground, but no similar conclusion has been obtained for the poor surrounding ground, and specific problems must be analyzed during construction.

## Introduction

**Figure 1:**
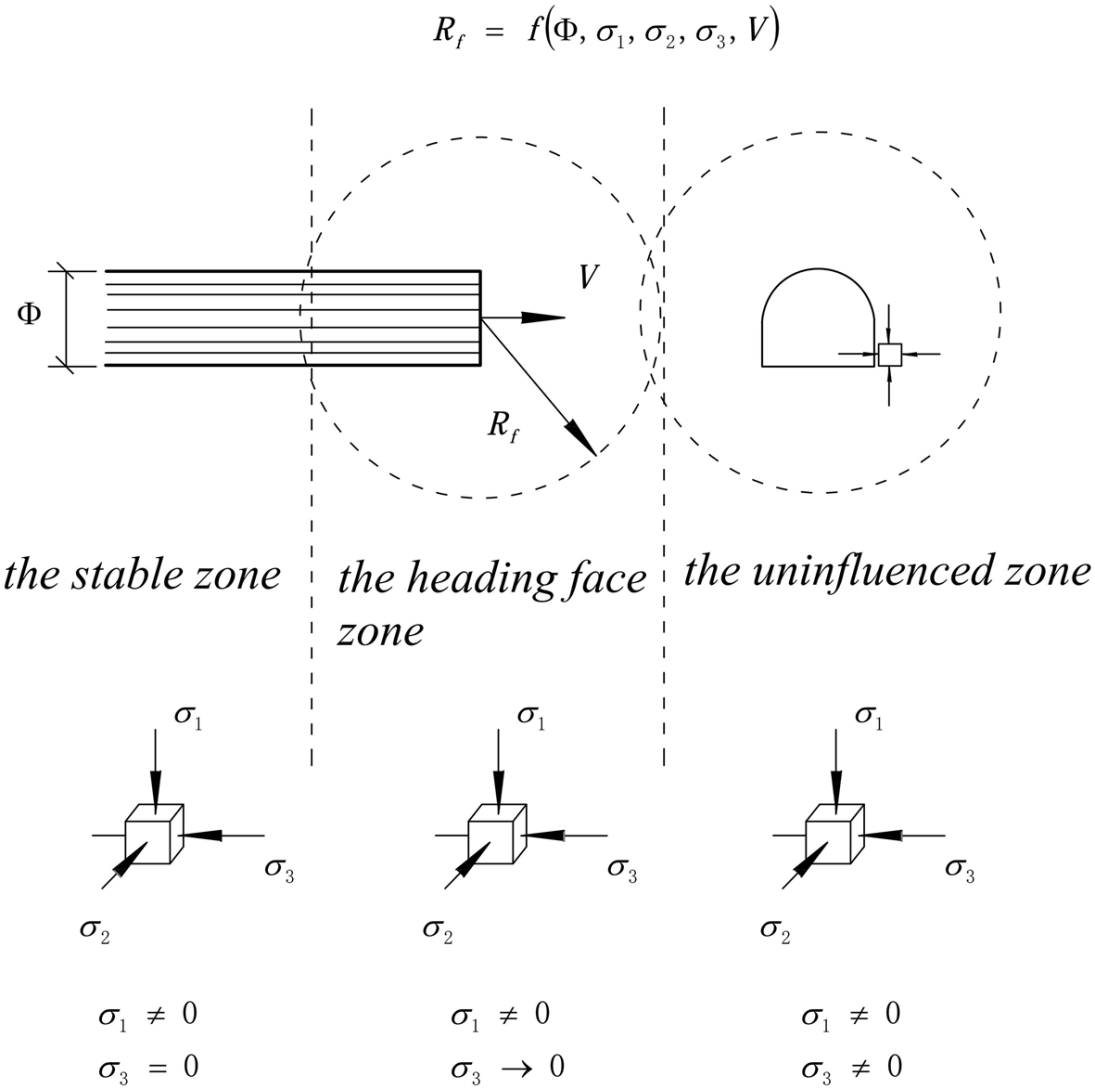
Typical zones of a tunnel^3^.

The construction of tunnels, particularly highway and railway tunnels, in soft surrounding ground often faces various challenges and difficulties^1^. According to the Analysis of Controlled Deformation in Rock and Soils (ADECO-RS)^2^, the soft surrounding ground of the tunnel can be classified into three distinctive zones^2^, as depicted in Fig. 1, in the uninfluenced zone, the surrounding ground is not impacted by excavation and is in a three-dimensional stress state. The heading face zone, also known as the transition zone, corresponds to the radius of influence of the face (*R*_*f*_). It consists of the tunnel face, the advanced core, and the cyclical footage. The excavation process greatly affects the stress state of the surrounding ground in this zone, causing it to transition from a three-dimensional stress state to a two-dimensional plane stress state. In the stable zone, the surrounding ground in a two-dimensional stress state tends to reach equilibrium.

According to a statistical analysis of tunnel collapse incidents^3^, 79% of accidents occurred in the heading face zone during construction, while the entrance collapse accounted for approximately 20%. It can be seen that the tunnel face of soft surrounding ground is at high risk of construction problems during the transition from a three-dimensional stress state to two-dimensional plane stress state. At present, scholars from various countries often utilize model experiments, limit equilibrium analysis, limit analysis, and numerical simulations to study the failure mechanisms and stability of the tunnel face in weak surrounding ground.Based on similarity theory, conducting experiments by reducing the prototype to a certain scale is a commonly used method in model tests^4^. The commonly employed model tests include centrifuge simulation tests and conventional gravity field tests^4^.Model tests provide an effective approach for studying tunnel face stability^6^, but they also have limitations and drawbacks^7^.

Limit equilibrium method is one of the methods for analyzing the stability of tunnel face in weak surrounding ground. It is based on constructing various hypothetical failure surfaces, such as plane^8^, polyline^9^, logarithmic spiral^10^, etc., dividing them into micro-elements and establishing differential balance equations, and finding the most dangerous sliding surface position from the selected failure forms.

Limit analysis can always obtain a more practical failure value by solving the upper and lower bound loads, no matter how complex the geometry and loading conditions of the soil and rock are^11^. In recent years, limit analysis has not only developed rapidly in slope stability, earth pressure calculation, foundation bearing capacity and other aspects, but also been applied and promoted in tunnel engineering^12^.

With the rapid advancement of computer technology, numerical methods based on continuous media, such as finite element, finite difference, boundary element^13^, as well as methods based on discontinuous media, such as discrete element, granular flow, and meshfree methods, have developed rapidly^14^. Concurrently, the numerical methods for solving limit analysis have become increasingly diverse, including variational methods, optimization methods, and numerical solution methods^15^.

However, most studies on tunnel face stability only focus on the transverse section, leaving limited research on the cyclical footage and advanced core in the three-dimensional longitudinal stability analysis. Furthermore, the Mohr–Coulomb strength criterion, which is widely used to study soil stability, does not take into account intermediate principal stress^16^, leading to results that are not consistent with reality. Therefore, based on the upper limit method and the twin shear unified strength theory^17^, a three-dimensional failure mechanism of the heading face zone considering the cyclic footage was established, providing a theoretical foundation for the reinforcement of the face and advanced core.

## Logarithmic spiral failure mechanism

Limit analysis has been applied to analyze the longitudinal stability of the tunnel face. Davis et al. (1980)^18^ established a failure mode with two blocks and three variables based on the principle of limit analysis. Leca & Bomreiux(1990)^19^ obtained two active failure modes and one passive failure mode by using a planar oblique truncated cone and axis-symmetric rotation around the cone center. Zhang et al. (2013) ^20^ constructed a new three-dimensional failure mechanism and discussed the longitudinal collapse mechanism of the vault based on a series of model test results and numerical analysis in cohesive soil.

Some scholars have noted that the number of cones in Leca and Dormieux failure modes is limited and not smooth, indicating a need for improved calculation accuracy. Soubra (2000)^21^ incorporated a logarithmic spiral curve section between two rigid inclined truncated cones to better match the tunnel face failure characteristics, which was then extended to shallow buried tunnels. Subrin & Wong (2002)^22^ assumed a logarithmic spiral surface in front of the tunnel face and used the upper bound limit analysis to calculate the tunnel face support force, which was smoother compared to Soubra's improved damage mechanism. Mollon et al.^23^ used a "point-to-point" spatial discrete technology to establish a failure mode composed of two logarithmic spiral curves and proposed the CSRSM method. Michalowski et al.^24^ constructed a logarithmic spiral cone with a shape similar to a trumpet. Based on the two-dimensional logarithmic spiral failure mode, Feng et al.^25^ constructed a three-dimensional failure mode.

**Figure 2:**
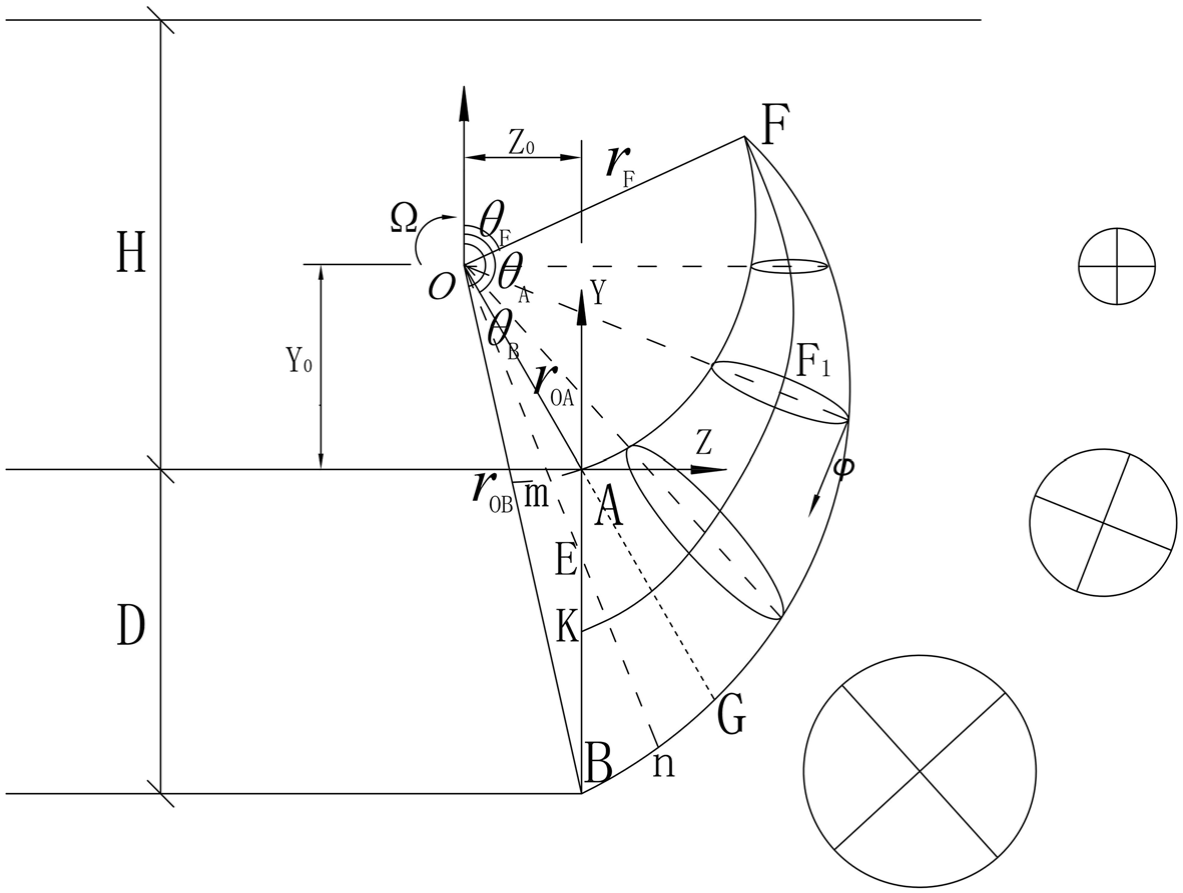
The double logarithmic spiral three-dimensional failure mode.

Currently, the stability analysis of tunnel faces generally does not consider the influence of cyclic footage and intermediate principal stress. Therefore, a three-dimensional failure mode and velocity field that take into account the cyclic footage and intermediate principal stress are reconstructed based on the logarithmic spiral failure mode, as shown in Fig. 2.

The logarithmic spiral three-dimensional failure mode is formed by rotating the distance between two logarithmic spiral curves as the diameter. In the figure, point **O** represents the center of rotation, while the distance from point **A** of the vault and point **B** of the bottom are, respectively,

1 $$r_{OA} = \sqrt {Z_{0}^{2} + Y_{0}^{2} } \,$$

2 $$\, r_{OB} = \sqrt {Z_{0}^{2} + (Y_{0} + D)^{2} }$$

**AF** and **BF** refer to the logarithmic spiral curves, which intersect at point **F**. The distance between point **F** and the rotation center point **O** is shown in the figure as **OF = **$r_{F}$.

3 $$r_{F} = \sqrt {r_{OA} r_{OB} \exp \left[ {\left( {\theta_{A} - \theta_{B} } \right)\tan \varphi } \right]}$$

The initial angle of the two logarithmic spiral curves is:

4 $$\theta_{A} = \pi - \tan^{ - 1} \left( { - \frac{{Z_{0} }}{{Y_{0} }}} \right) \,$$

5 $$\theta_{B} = \pi - \tan^{ - 1} \left( { - \frac{{Z_{0} }}{{Y_{0} + D}}} \right)$$

Then the angle of $\theta_{F}$ is:

6 $$\theta_{F} = \left[ {\theta_{A} + \theta_{B} - \frac{{\ln \left( {{\raise0.7ex\hbox{${r_{OB} }$} \!\mathord{\left/ {\vphantom {{r_{OB} } {r_{oA} }}}\right.\kern-0pt} \!\lower0.7ex\hbox{${r_{oA} }$}}} \right)}}{\tan \varphi }} \right]/2$$

In limit analysis, the velocity field of frictional materials must comply with the associated flow rules, and the angle between the velocity and the tangent line on the discontinuity surface should match the internal friction angle of the soil.

Then, for the double logarithmic spiral failure mode, the expression for the double logarithmic spiral line can be obtained when any point in the sliding region rotates around the **O** point with a constant angular velocity Ω, given by:

7 $$\, r_{AF} = r_{oa} \exp \left[ {\left( {\theta_{A} - \theta } \right)\tan \varphi } \right]$$

8 $$r_{BF} = r_{ob} \exp \left[ {\left( {\theta - \theta_{B} } \right)\tan \varphi } \right]$$

## The upper limit solution

### Two-shear unified strength theory

Since the Coulomb theory was proposed in 1773, various strength theories for rock and soil materials have been developed, each with a certain scope of application. Based on a unified physical model, Yu et al.^26^ proposed the unified friction angle and unified cohesion parameters using the twin shear unified strength theory, which are:

9 $$\sin \varphi_{t} = \frac{{b\left( {1 - m} \right) + \left( {2 + b + bm} \right)\sin \varphi_{0} }}{{2 + b\left( {1 + \sin \varphi_{0} } \right)}}$$

10 $$c_{t} = \frac{{2\left( {1 + b} \right)c_{0} \cos \varphi_{0} }}{{2 + b\left( {1 + \sin \varphi_{0} } \right)}} \times \frac{1}{{\cos \varphi_{t} }}$$

In the Eq. (9), ***m*** represents the intermediate principal stress coefficient, which is generally taken as 1 unless otherwise specified. The parameter ***b*** is a weighted parameter that reflects the influence of both the intermediate shear stress and the normal stress on the corresponding action surface with respect to the yield or failure of the material.

### The upper limit analysis without considering the cyclical footage

#### Gravity work

**Figure 3:**
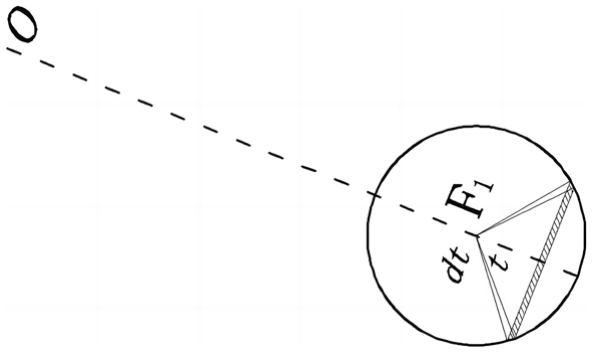
The circular cross-section of the three-dimensional region **AFG**.

When $\theta_{F} < \theta < \theta_{A}$, circles of different shapes can be obtained by intersecting a cone with a plane passing through the point **O** in different relative positions, as shown in Fig. 3, the distance $r_{FK}$ from the center of the circular cross-section to the rotation center point **O** is determined as:

11 $$r_{FK} = (r_{AF} + r_{BF} )/2$$

The radius of the circular cross-section is:

12 $$R_{FK} = (r_{BF} - r_{AF} )/2$$

The shaded area in Fig. 3 is:

13 $$ds = R_{FK}^{2} \left( {1 - \cos 2t} \right)dt$$

The distance between the barycenter of the shadow area and the rotation center **O** is:

14 $$dl = r_{FK} + R_{FK} \cos t$$

The shaded volume of the micro-unit is:

15 $$dV = \left( {r_{FK} + R_{FK} \cos t} \right)d\theta R_{FK}^{2} \left( {1 - \cos 2t} \right)dt$$

The velocity of the micro-unit in the shaded part is:

16 $$dv = \left( {r_{FK} + R_{FK} \cos t} \right)\Omega$$

Then the gravitational work done by the micro-unit in the shaded part is:

17 $$dW_{1} = \gamma \left( {r_{FK} + R_{FK} \cos t} \right)\Omega \cos \left( {\theta - \frac{\pi }{2}} \right)\left( {r_{FK} + R_{FK} \cos t} \right)d\theta R_{FK}^{2} \left( {1 - \cos 2t} \right)dt$$

When $\theta_{F} < \theta \le \theta_{A}$,$0 \le t \le \pi$,thus the gravitational work of three-dimensional region **AFG** is:

18 $$W_{1} = \int_{{\theta_{F} }}^{{\theta_{A} }} {\int_{0}^{\pi } {\gamma \left( {r_{FK} + R_{FK} \cos t} \right)\Omega \cos \left( {\theta - \frac{\pi }{2}} \right)\left( {r_{FK} + R_{FK} \cos t} \right)d\theta R_{FK}^{2} \left( {1 - \cos 2t} \right)dt} }$$

**Figure 4:**
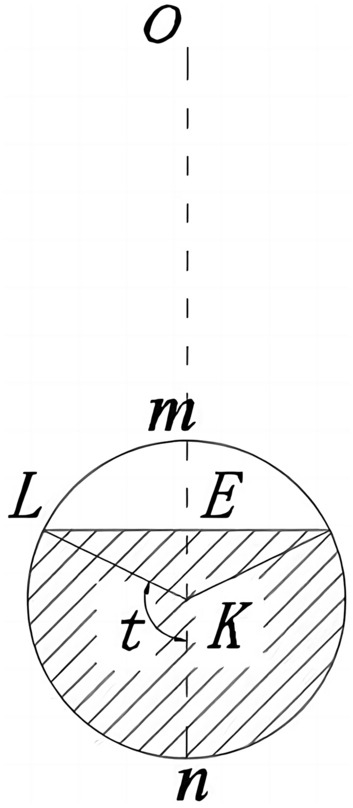
ABG section circle in 3D area.

When $\theta_{A} < \theta \le \theta_{B}$, the three-dimensional region **ABG** is intercepted by the plane as shown in Fig. 4. The plane is intercepted by a circle that is not complete, and the geometric relationship is shown as:

19 $$\frac{OE}{{\sin \theta_{A} }} = \frac{AE}{{\sin \left( {\theta - \theta_{A} } \right)}} = \frac{{r_{OA} }}{{\sin \left( {\pi - \theta } \right)}} \Rightarrow OE = \frac{{r_{OA} \sin \theta_{A} }}{{\sin \left( {\pi - \theta } \right)}}$$

The variation of the angle ***t*** can be obtained in the triangle **LEK**

20 $$\cos \left( {\pi - t} \right) = \frac{{r_{FK} }}{{R_{FK} }} - \frac{{r_{OA} \sin \theta_{A} }}{{R_{FK} \sin \left( {\pi - \theta } \right)}} \Rightarrow t_{\max } = \arccos \left( {\frac{{r_{OA} \sin \theta_{A} }}{{R_{FK} \sin \left( {\pi - \theta } \right)}} - \frac{{r_{FK} }}{{R_{FK} }}} \right)$$

Then the work done by gravity in the area **ABG**

21 $$W_{2} = \int_{{\theta_{A} }}^{{\theta_{B} }} {\int_{0}^{{t_{\max } }} {\gamma \left( {r_{FK} + R_{FK} \cos t} \right)\Omega \cos \left( {\theta - \frac{\pi }{2}} \right)\left( {r_{FK} + R_{FK} \cos t} \right)d\theta R_{FK}^{2} \left( {1 - \cos 2t} \right)dt} }$$

#### Work of the external force

**Figure 5:**
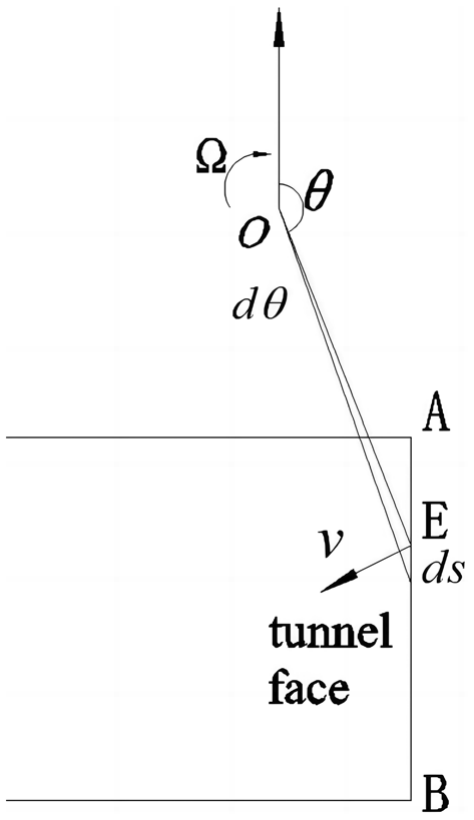
Schematic diagram of the force on tunnel face.

In order to facilitate calculation, it is assumed that the supporting force of the tunnel face is evenly distributed, and the velocity direction of the tunnel face **AB** varies with the angle $\theta$, as shown in Fig. 5, in the circle the length of **AE** can be expressed as:

22 $$AE = \frac{{r_{OA} \sin \left( {\theta - \theta_{A} } \right)}}{\sin \theta }$$

The angle $\theta$ derivative of **AE** is obtained as:

23 $$\frac{dAE}{{d\theta }} = \frac{{r_{OA} \sin \theta_{A} }}{{\sin^{2} \theta }}$$

As shown in Fig. 4 and Fig. 5, the **EL** length perpendicular to the paper surface is:

24 $$EL = \sqrt {R_{FK}^{2} - \left( {r_{FK} - \frac{{r_{OA} \sin \theta_{A} }}{{\sin \left( {\pi - \theta } \right)}}} \right)^{2} }$$

The micro-unit area $dS$ of the tunnel-face is:

25 $$dS = 2ELdAE = 2EL\frac{{r_{OA} \sin \theta_{A} }}{{\sin^{2} \theta }}d\theta = 2EL\frac{OE}{{\sin \theta }}d\theta$$

The micro unit speed is:

26 $$v = OE\Omega$$

Then the work done by the support force on the tunnel-face is:

27 $$P_{T} = \int_{{\theta_{A} }}^{{\theta_{B} }} {dP_{T} } = \int_{{\theta_{A} }}^{{\theta_{B} }} {\sigma_{t} dSv\sin \left( {\theta - \frac{\pi }{2}} \right)} = \int_{{\theta_{A} }}^{{\theta_{B} }} {\sigma_{t} 2EL\frac{OE}{{\sin \theta }}d\theta OE\Omega \sin \left( {\theta - \frac{\pi }{2}} \right)}$$

#### Energy dissipation analysis

**Figure 6:**
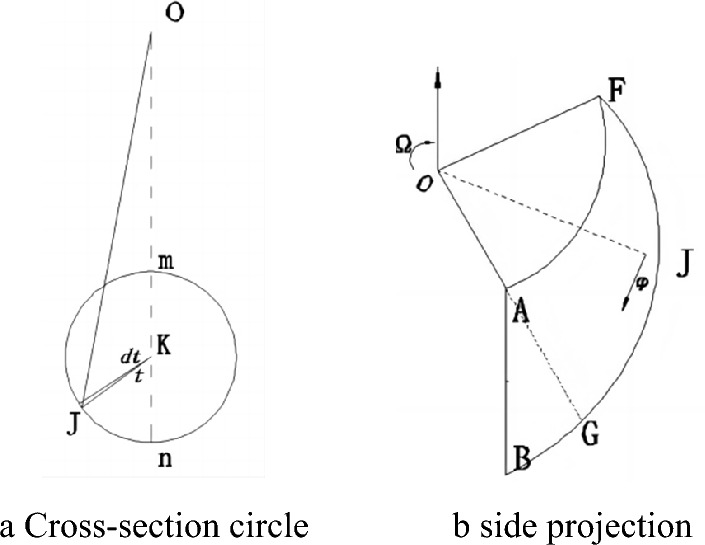
Diagram of calculation of energy dissipation.

Velocity variations and energy dissipation occur at the velocity discontinuity. In the three-dimensional logarithmic spiral failure mode, energy dissipation is caused by the discontinuity between the side of the **AFG** area and the side of **ABG**, as shown in Fig. 6.

When solving for the energy dissipation on the velocity discontinuity of the **AFG** region, it is necessary to solve for the side area of the **AFG** region first. According to the geometric relationship shown in Fig. 6, the distance from the center of rotation **O** to any position on the logarithmic helix can be obtained by taking the free angle $\theta$ and central angle ***t***.

28 $$OJ^{2} = r_{{_{FK} }}^{2} + R_{{_{FK} }}^{2} - 2r_{FK} R_{FK} \cos \left( {\pi - t} \right)$$

As shown in Fig. 6a, when the angle of the center of the circle is $dt$ in arbitrary cross-sectional circle, the arc length is $dl = R_{FK} dt$, corresponding to the arc height $dh = {{OJd\theta } \mathord{\left/ {\vphantom {{OJd\theta } {\cos \varphi }}} \right. \kern-0pt} {\cos \varphi }}$, and the velocity is $v = OJ\Omega \cos \varphi$. the total internal energy dissipation of velocity discontinuity integration of the **AFG** is:

29 $$S_{1} = \int_{{\theta_{F} }}^{{\theta_{A} }} {\int_{0}^{\pi } {2R_{FK} dt\frac{OJd\theta }{{\cos \varphi }}OJ\Omega \cos \varphi c} } = \int_{{\theta_{F} }}^{{\theta_{A} }} {\int_{0}^{\pi } {2R_{FK} OJ^{2} \Omega c} } d\theta dt$$

In which,

30 $$t_{\max } = \arccos \left( {\frac{{r_{OA} \sin \theta_{A} }}{{\sin \left( {\pi - \theta } \right)R_{FK} }} - \frac{{r_{FK} }}{{R_{FK} }}} \right)$$

Similarly, the total dissipation energy of region **ABG** can be obtained as:

31 $$S_{2} = \int_{{\theta_{A} }}^{{\theta_{B} }} {\int_{0}^{{t_{\max } }} {2R_{FK} OJ^{2} \Omega c} } d\theta dt$$

### Upper limit analysis considering cyclical footage

**Figure 7:**
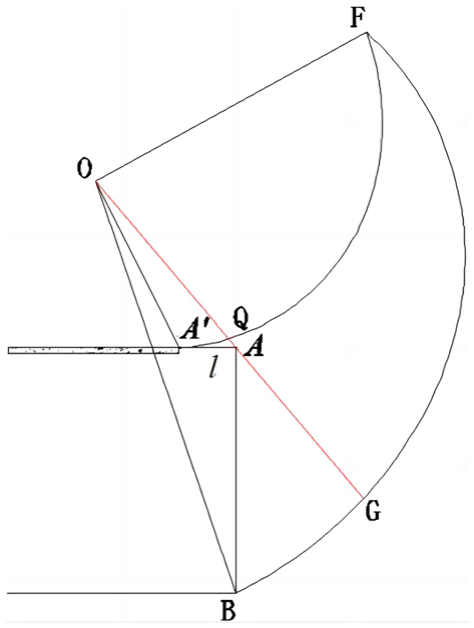
Three-dimensional logarithmic spiral failure mode considering cyclical footage.

The cyclical footage ***l*** is presented in the three-dimensional logarithmic spiral failure mode, as shown in Fig. 7. According to existing studies^27–30^, tunnel vault failure mainly occurs in the **A**^***'***^**AQ** region along the longitudinal direction of the tunnel. By comparing Fig. 2 with Fig. 7, it can be seen that the starting point of the logarithmic spiral curve changes from point **A** to point **A**^***'***^. Similar to Eqs. (1) through (8), the logarithmic spiral radius considering the cyclical footage is given by:

32 $$r_{{OA^{\prime}}} = \sqrt {\left( {Z_{0} + l} \right)^{2} + Y_{0}^{2} } \,$$

33 $$r^{\prime}_{F} = \sqrt {r_{{OA^{\prime}}} r_{OB} \exp \left[ {\left( {\theta_{{A^{\prime}}} - \theta_{B} } \right)\tan \varphi } \right]}$$

The initial angle is:

34 $$\theta_{{A^{\prime}}} = \pi - \tan^{ - 1} \left( { - \frac{{Z_{0} + l}}{{Y_{0} }}} \right) \,$$

35 $$\theta^{\prime}_{F} = [\theta_{{A^{\prime}}} + \theta_{B} - \frac{{\ln ({\raise0.7ex\hbox{${r_{OB} }$} \!\mathord{\left/ {\vphantom {{r_{OB} } {r_{{oA^{\prime}}} }}}\right.\kern-0pt} \!\lower0.7ex\hbox{${r_{{oA^{\prime}}} }$}})}}{\tan \varphi }]/2$$

The logarithmic spiral **A**^**'**^**F** is expressed as:

36 $$\, r_{{A^{\prime}F}} = r_{{OA^{\prime}}} \exp \left[ {\left( {\theta_{{A^{\prime}}} - \theta } \right)\tan \varphi } \right]$$

The distance from the center of the cross-sectional circle to the center of rotation **O** also changes, i.e.

37 $$r_{FK}^{\prime } = (r_{{A^{\prime } F}} + r_{BF} )/2$$

38 $$R_{FK}^{\prime } = \left( {r_{BF} - r_{A\prime F} } \right)/2$$

#### Work done by external forces

In terms of the work done by gravity, the work of the three-dimensional logarithmic spiral is composed of two parts: the **QGF** and **ABG** regions. It should be noted that the gravitational work done in the **A**^***'***^**AQ** region is not included in the calculation because it is a static equilibrium problem and the work done is relatively small.

Firstly, the **QGF** region is similar to the **AGF** region when the cyclic footage is not considered, by comparing Fig. 6 with Fig. 7, it is necessary to pay attention to the changes of parameters in the Eq. (18), which are as follows:

39 $$W_{1}^{\prime } = \int_{{\theta_{F} }}^{{\theta_{A} }} {\int_{0}^{\pi } {\gamma \left( {r_{FK}^{\prime } + R_{FK}^{\prime } \cos t} \right)\Omega \cos \left( {\theta - \frac{\pi }{2}} \right)\left( {r_{FK}^{\prime } + R_{FK}^{\prime } \cos t} \right)d\theta R_{FK}^{\prime 2} \left( {1 - \cos 2t} \right)dt} }$$

Secondly, the work done by gravity in the **ABG** region is:

40 $$t_{\max }^{\prime } = \arccos \left( {\frac{{r_{{OA^{\prime } }} \sin \theta_{A} }}{{\sin \left( {\pi - \theta } \right)R_{FK}^{\prime } }} - \frac{{r_{FK}^{\prime } }}{{R_{FK}^{\prime } }}} \right)$$

41 $$W_{2}^{\prime } = \int_{{\theta_{A} }}^{{\theta_{B} }} {\int_{0}^{{t_{\max }^{\prime } }} {\gamma \left( {r_{FK}^{\prime } + R_{FK}^{\prime } \cos t} \right)\Omega \cos \left( {\theta - \frac{\pi }{2}} \right)\left( {r_{FK}^{\prime } + R_{FK}^{\prime } \cos t} \right)d\theta R_{FK}^{\prime 2} \left( {1 - \cos 2t} \right)dt} }$$

#### Internal energy dissipation

According to the Eq. (29) deducing, the regional **QFG** dissipation energy can be quickly derived as:

42 $$S^{\prime}_{1} = \int_{{\theta_{F} }}^{{\theta_{{A^{\prime}}} }} {\int_{0}^{\pi } {2R^{\prime}_{FK} dt\frac{OJd\theta }{{\cos \varphi }}OJ\Omega \cos \varphi c} } = \int_{{\theta_{F} }}^{{\theta_{{A^{\prime}}} }} {\int_{0}^{\pi } {2R^{\prime}_{FK} OJ^{2} \Omega c} } d\theta dt$$

Similarly, the dissipation energy of region **ABG** is:

43 $$S^{\prime}_{2} = \int_{{\theta_{{A^{\prime}}} }}^{{\theta_{B} }} {\int_{0}^{{t^{\prime}_{\max } }} {2R^{\prime}_{FK} OJ^{2} \Omega c} } d\theta dt$$

When the external energy dissipation and the internal energy dissipation are equal, the whole logarithmic spiral mechanism is in a limit equilibrium state, the ultimate support force of the tunnel face can be obtained as follows:

44 $$W_{1} { + }W_{2} - P_{T} - S_{1} - S_{2} = 0$$

45 $$W^{\prime}_{1} { + }W^{\prime}_{2} - P_{T} - S^{\prime}_{1} - S^{\prime}_{2} = 0$$

### Contrast studies

**Figure 8:**
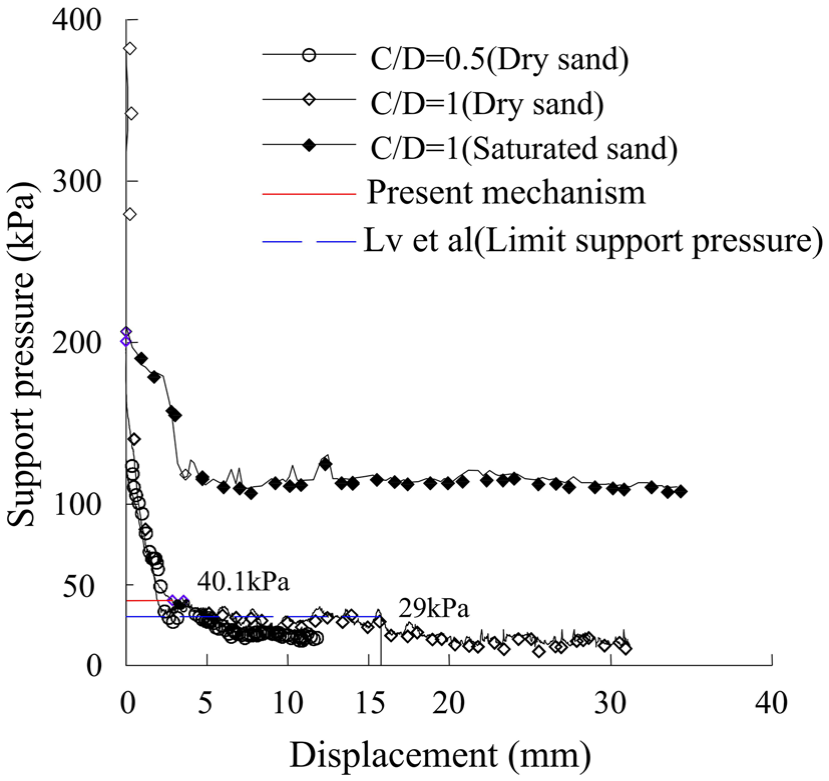
Curves of support stress and displacement under Centrifugal test^31^.

Lv^31^ obtained the supporting stress of sand and saturated sand based on centrifugal model test, as shown in Fig. 8, C/D is the ratio of the tunnel depth to diameter. At the initial displacement stage of the tunnel face center point (indicated from 0 to 3 mm), the supporting stress drops from 140 kPa to about 30kPa, and the difference in ultimate supporting force is small under different buried depths. Through centrifugal simulation and numerical analysis, Lv^31^ calculated that the ultimate support force of the tunnel face in the case of dry sand was 29 kPa.

According to the law of similarity, in the Lv’s^31^ tunnel model test, the diameter of the tunnel was equivalent to 15 m, and the density,$\gamma$, the cohesion,$c$ and the internal friction angle $\phi$ of the surrounding ground are 1.7 g/cm^3^, 4.5 kPa and 35°, respectively. Substituting these values into Eq. (44), the tunnel-face support force was calculated to be 40.1 kPa, as shown in Fig. 8, and the displacement of the center point of the tunnel face was about 2.5 mm in the centrifugal simulation test. Therefore, the support force of the tunnel-face obtained by the three-dimensional logarithmic spiral mode is safer and the displacement is smaller, which fully utilizes the load-bearing capacity of the surrounding ground itself and is more consistent with the actual situation.

## Parametric study

### The relation between internal friction angle and cyclical footage

**Figure 9:**
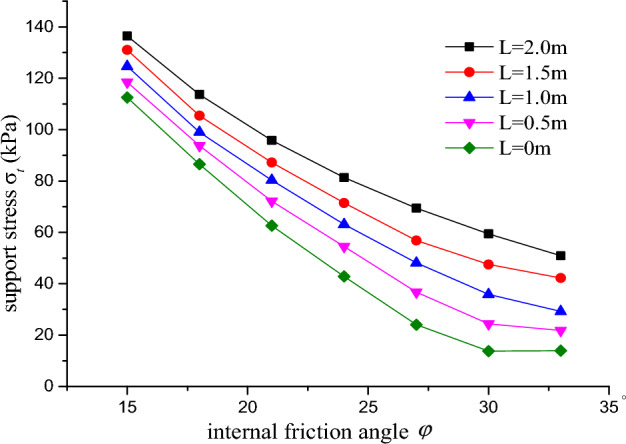
Curve of support stress and internal friction angle.

When the diameter of the tunnel, the density and the cohesion are 15 m, 1.8 g/cm^3^, 4.5 kPa, respectively, the curve of the support stress and internal friction angle for various cyclical footage is shown in Fig. 9.

As shown in Fig. 9, the support pressure of tunnel face gradually decreases with the increase of internal friction angle when cyclical footage is 2 m. Note that the decreasing amplitude of the support pressure of tunnel face is relatively gentle when the internal friction angle is greater than 30°, especially it is more gentle than others when cyclical footage is not taken into account. Then it is uneconomic to use physical reinforcement measures to improve the internal friction angle, such as anchor rod, advance small pipe.

At the same time, with the increase of cyclical footage, the supporting stress required by the tunnel face also increases synchronously. When the internal friction angle is equal to 30°, the increase amplitude is the largest, indicating that the cyclical footage has a great influence on the excavation.

### The relation between support stress and cohesion

When the diameter of the tunnel, the density and the internal friction angle are 15 m, 1.8 g/cm^3^, 25°.

**Figure 10:**
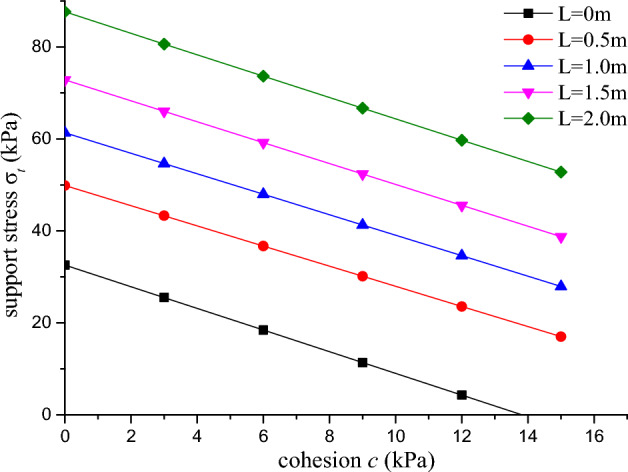
Support pressure and cohesion diagram.

In different cyclical footage, the relationship between support force and cohesion can be observed as illustrated in Fig. 10.

As can be gleaned from the Fig. 10, as cohesion increases, the support pressure on the tunnel surface decreases. The rate of decrease in support pressure is largely consistent across different cyclical footage, however, the magnitude of support pressure exhibits variations depending on the cyclical footage. At a cyclical footage of 0, the required support pressure on the tunnel surface is the least, and when cohesion approaches 13.8 kPa, the tunnel surface is deemed stable. As the cyclical footage increases to 0.5 m, it can be observed from the figure that the required support pressure on the tunnel surface increases to 20 kPa. Between cyclical footage of 0.5 m and 1.5 m, the increase in support pressure on the tunnel surface is relatively small with respect to the increase in cyclical footage, indicating that the ultimate support force of the tunnel surface is affected by the cyclical footage of the tunnel surface and the initial effect is more pronounced. Therefore, particular attention should be paid to monitoring the initial excavation process of the excavation cycle in construction and observing the deformation of the surrounding ground.

### Relationship between support force and cyclical footage

**Figure 11:**
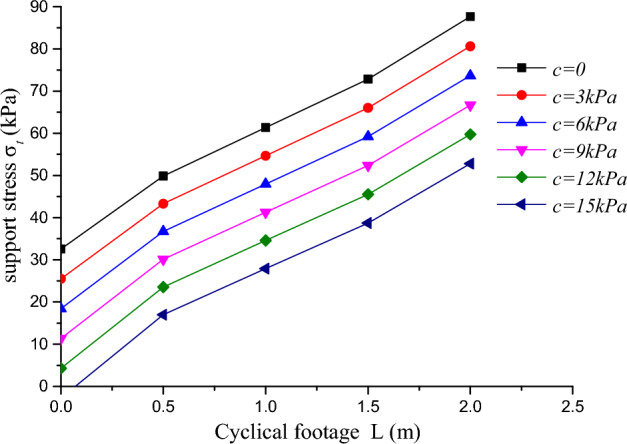
Relationship between support force and cycle length under different cohesion.

When the tunnel diameter is 15 m and the unit weight is 18kN/m^3^, with a weighted parameter ***b*** = 0, the relationship between the support pressure and the cycle advance under different cohesion is shown in Fig. 11.

From the Fig. 11, it can be seen that as the cyclical footage increases, the support pressure required at the tunnel face increases simultaneously, but the initial increase trend is greater than the mid-term increase trend, and the mid-term increase trend is smaller than the later increase trend, which is consistent with the situation in Fig. 10 and reflects the potential for optimization of the impact of the cyclical footage on the tunnel face excavation in the tunnel. When the cyclical footage is less than 0.5 m, its impact on the tunnel face is the greatest, at which point the stress characteristics of the tunnel face region change from a three-dimensional stress state to a two-dimensional stress state, and the required support pressure for the tunnel face rapidly increases. As the cyclical footage continues to increase, its impact on the tunnel face gradually decreases and has similar characteristics for different cyclical footage, which can guide similar construction treatment methods for this section in construction. When the cyclical footage increases to 2 m, the superposition of horizontal and vertical failure trends in the unsupported section of the tunnel face increases, and the increase rate of support pressure for the tunnel face increases, reflecting the more complex stress situation of the roof face region, and the simplified analysis of the vertical failure trend can no longer be valid.

### Effect of weighting factor

**Figure 12:**
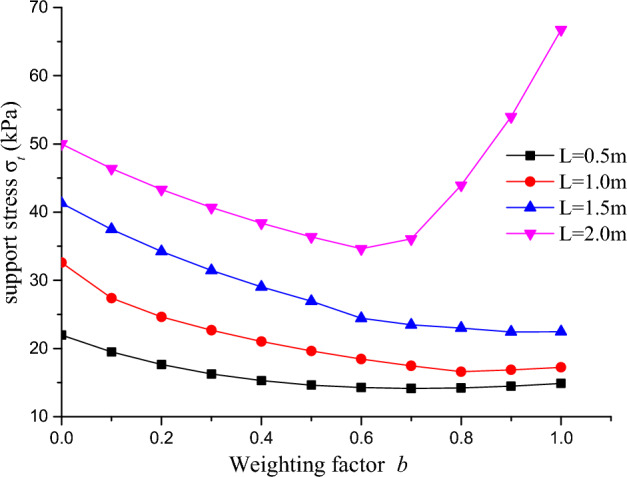
Relationship between support force and weighting factor under different cyclical footage ($\varphi = 25^{0}$).

**Figure 13:**
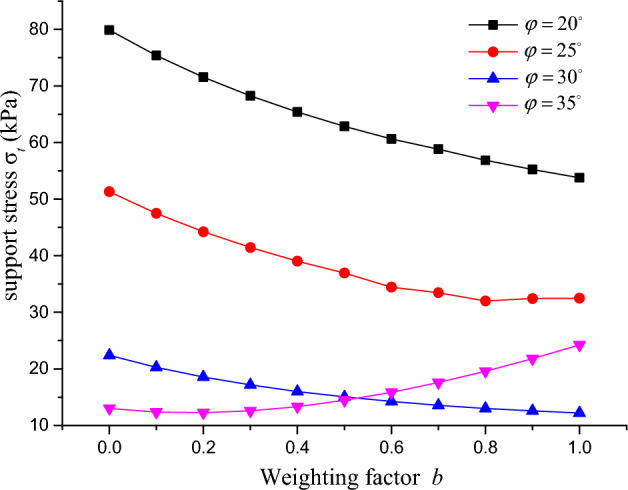
Relationship between support pressure and weighting factor.

**Figure 14:**
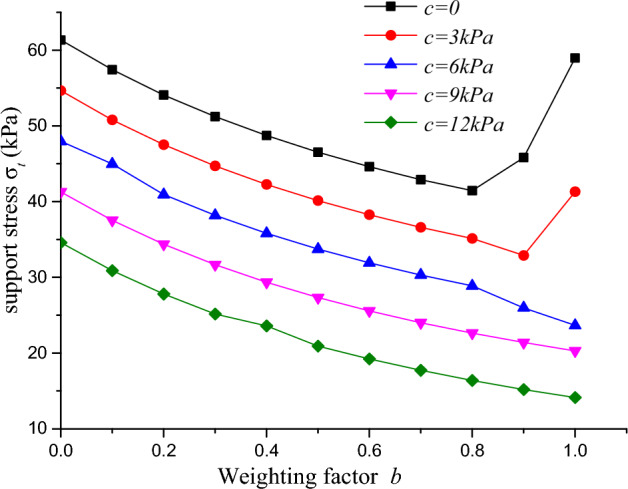
Relationship between support pressure and weighting factor under different cohesive stresses.

Based on the research findings of Yu's^26^ theory, the influence of the intermediate principal stress is considered using the unified strength theory, and the weighted factor ***b*** is introduced to analyze the changing laws of tunnel face supporting pressure under the three-dimensional logarithmic spiral failure mode, as shown in Fig. 12, 13, 14.

The Fig. 12 reflects the relationship between supporting force and weighting factor under different cyclical footage. It can be seen from the figure that when the cyclical footage is less than or equal to 1.5 m, the tunnel face supporting pressure gradually decreases with the increase of the weighting factor, reflecting the positive embedding effect of the intermediate principal stress. However, with the increase of the weighting factor, the decrease of the supporting pressure becomes smaller, reflecting that there may be a stable interval of supporting pressure under different criteria. When the cyclical footage is greater than 2 m, the supporting pressure shows different patterns with the change of the weighting factor, as can be seen from the Fig. 12. When the weighting factor ***b*** is less than 0.6, the tunnel face supporting pressure decreases with the increase of the weighting factor. But when the weighting factor is greater than 0.6, the supporting pressure of the tunnel face gradually increases with the increase of the weighting factor, indicating that the increase of the cyclical footage will cause a large-area unsupported surface in the tunnel, changing the stress characteristics of the tunnel face region and reducing the constraint of the intermediate principal stress on the cyclical footage, making the tunnel face region unstable, similar to the pattern reflected in Fig. 11.

The relationship between the tunnel surface support pressure and the weighting factor under different internal friction angles is reflected in Fig. 13. As seen from the figure, when the internal friction angle is less than 30°, the tunnel surface support pressure gradually decreases as the weighting factor increases. As the weighting factor approaches 1, the decrease is less noticeable, indicating a weakened positive effect of the intermediate principal stress. However, when the internal friction angle is greater than 35°, the tunnel surface support pressure gradually increases with the increase in the weighting factor, and the growth is greater as it approaches 1. This suggests that the impact of the intermediate principal stress on the tunnel surface support pressure is not always positive and has a negative impact on the stability of the tunnel surface area.

With a constant internal friction angle, the relationship between the support pressure of the tunnel surface and the weighting factor under different cohesive stresses can be obtained, as shown in Fig. 14. When the cohesive stress is greater than 6 kPa, the support pressure of the tunnel surface gradually decreases with the increase of the weighting factor. When the cohesive stress is less than 6 kPa, the change trend of the support pressure of the tunnel surface with the weighting factor is different. After the weighting factor is greater than 0.8, the support pressure increases with the increase of the weighting factor, and the faster the growth rate is, the smaller the cohesive stress is. Combining the analysis of Fig. 13, it can be seen that when the internal friction angle is larger (above 35°) and the cohesive stress is smaller (less than 6 kPa), the extra growth of the support pressure of the tunnel surface will cause adverse impact on the stability of the tunnel face area under the joint action of the intermediate principal stress.

## Conclusions

Based on limit analysis, the logarithmic spiral three-dimensional failure mode considering the cyclical footage is constructed by introducing the dual-shear unified strength theory proposed by Yu^26^. The corresponding calculation equation was derived based on the upper bound theory and the impact of relevant parameters on the stability of the tunnel face area was analyzed. The main research conclusions are as follows:

(1) The internal friction angle and cohesion have different levels of impact on the stability of the tunnel face. When the internal friction angle is less than 30°, it is advisable to prioritize improving the internal friction angle performance of the surrounding ground mass near the tunnel face to enhance its stability. On the other hand, when the internal friction angle is larger, especially above 30°, it is better to prioritize construction methods that enhance cohesion, improve the cohesion performance of the surrounding ground mass, and enhance overall stability.

(2) In construction, special attention should be paid to the initial excavation process of the tunnel's cyclical footage, paying attention to observe the deformation of the surrounding ground. At the same time, the growth of the cyclical footage does not infinitely increase the supporting pressure of the tunnel face, and there are limitations in the impact on the stability of the tunnel face.

(3) The dual-shear unified strength theory can be applied in good surrounding ground, resulting in improved force distribution in the soil mass and more favorable impact of the intermediate principal stress on the stability of the tunnel face, indicating that the Mohr–Coulomb criterion tends to be conservative in relatively favorable soil conditions.

(4) In the unified strength theory, there is a significant difference in the impact of cohesive stress and internal friction angle on the stability of the tunnel face. The improvement of cohesive stress is more stable, while the improvement of internal friction angle varies greatly. When the internal friction angle is large (above 35°) and the cohesive stress is small (less than 6 kPa), the use of equivalent Mohr–Coulomb strength is not beneficial for the stability of the tunnel face area.

## Data Availability

Data will be available by the corresponding author on reasonable request.
